# Arbuscular mycorrhizal fungi affect the expression of *PxNHX* gene family, improve photosynthesis and promote *Populus simonii*×*P. nigra* growth under saline-alkali stress

**DOI:** 10.3389/fpls.2023.1104095

**Published:** 2023-01-30

**Authors:** Fengxin Dong, Yihan Wang, Jing Tao, Tingying Xu, Ming Tang

**Affiliations:** ^1^ College of Forestry, Northwest A&F University, Xianyang, China; ^2^ Boone Pickens School of Geology, Oklahoma State University, Stillwater, OK, United States; ^3^ Guangdong Laboratory for Lingnan Modern Agriculture, College of Forestry and Landscape Architecture, South China Agricultural University, Guangzhou, China

**Keywords:** saline-alkali stress, arbuscular mycorrhizal fungi, *Populus simonii×P. nigra*, *NHX* gene family, *Funneliformis mosseae*

## Abstract

**Introduction:**

Saline-alkali stress seriously endangers the normal growth of *Populus simonii*×*P. nigra*. Arbuscular mycorrhizal (AM) fungi can enhance the saline-alkali tolerance of plants by establishing a symbiotic relationship with them.

**Methods:**

In this study, a pot experiment was conducted to simulate a saline-alkali environment where *Populus simonii*×*P. nigra* were inoculated with *Funneliformis mosseae* to explore their effects on the saline-alkali tolerance of *Populus simonii*×*P. nigra*.

**Results and Discussion:**

Our results show that a total of 8 *NHX* gene family members are identified in *Populus simonii*×*P. nigra*. *F. mosseae* regulate the distribution of Na+ by inducing the expression of *PxNHXs*. The pH value of poplar rhizosphere soil is reduced, result in the promote absorption of Na^+^ by poplar, that ultimately improved the soil environment. Under saline-alkali stress, *F. mosseae* improve the chlorophyll fluorescence and photosynthetic parameters of poplar, promote the absorption of water, K^+^ and Ca^2+^, thus increase the plant height and fresh weight of aboveground parts, and promote the growth of poplar. Our results provide a theoretical basis for further exploring the application of AM fungi to improve the saline-alkali tolerance of plants.

## 1 Introduction

About 1×10^9^ hm^2^ of saline-alkali soil is distributed all over the world (Liu et al., 2022), which seriously restricts the development of agricultural economy. Influenced by natural factors such as climate change and human factors such as improper irrigation methods, the saline-alkali region continues to expand from year to year (Evelin et al., 2019). China’s existing saline-alkali land is about 3.6×10^7^ hm^2^, mainly distributed in the northeast, northwest and coastal areas of north China (Lim et al., 2017; Yang et al., 2019b). Currently, the ecological environment of these areas is being improved by implementing the Three-North Shelter Forest Project. *Populus simonii*×*P. nigra* as a hybrid of *P. simonii* and *P. nigra* with the unique feature of elevated saline-alkali tolerance has been currently widely used in the Three-North Shelter Forest Project (Zhang, 2020). However, problems still exist before widely applying *Populus simonii*×*P. nigra* for environmental remediations such as low survival rate and moderate growth rate of seedlings after transplanting.

The damage of saline-alkali stress to plants is multifaceted. On the one hand, the accumulation of excess Na^+^ in cells can cause ion toxicity and physiological drought, leading to ABA-induced stomatal closure, limiting the entry of carbon dioxide into leaves, reducing the net photosynthetic rate and transpiration efficiency, thus limiting the normal growth of plants (Mohamed et al., 2020). At the same time, the content of reactive oxygen species can exceed the threshold, destroy the biofilm such as cell membrane and thylakoid membrane, degrade chlorophyll, and inhibit the activity of PS I and PS II (Zhang et al., 2020b). On the other hand, excessive ${\text{CO}}_{3}^{2-}$ can cause the precipitation of Ca^2+^ and Mg^2+^ by forming insoluble carbonate minerals in the soil, which reduces the bioavailability of these nutrient ions by the root system (Hui et al., 2022). Therefore, effective actions need to be taken to help plants alleviate the negative effects from saline-alkali stress.

Arbuscular mycorrhizal (AM) fungi can establish symbiotic relationships with more than 80% of terrestrial plants and are widely distributed in saline-alkali soils; they are considered as a biological tool capable to enhance plant saline-alkali tolerance (Smith and Read, 2008; Evelin et al., 2019; Wang et al., 2022b). Studies have shown that AM fungi can improve plants’ saline-alkali tolerance by promoting plant’s absorption of nutrients and water, improving rhizosphere soil conditions, improving plant photosynthesis, and inducing the expression of genes, such as Na^+^/H^+^ antiporter genes (NHX) (Smith et al., 2009; Evelin et al., 2019). For example, Kong et al. (2019) found that inoculating AM fungi in *Lycopersicon esculentum* Mill. under saline-alkali stress can increase chlorophyll concentration, net photosynthetic rate, stomatal conductance and transpiration rate. Yang et al. (2019a) found that inoculation with *Funneliformis mosseae* can increase *Pyrus betulaefolia* Bunge’s biomass and K^+^/Na^+^ ratio under saline-alkali stress. Although *Populus* is a class of plants dependent on mycorrhizal symbiosis, it takes a long time to establish a high degree of symbiotic relationship with AM fungi under normal, natural conditions (Yuan et al., 2019). Thus, inoculating the hybrid poplar species *P. simonii*×*P. nigra* with AM fungi may highly be possible to improve its saline-alkali tolerance. In addition, most current research specifically focused on the effects of AM fungi on plants under salt stress, less attention has been paid to the effects of AM fungi on plants under saline-alkali stress. Thus, detailed investigation of the role of AM fungi on populus under saline-alkali stressed environment is needed.

In order to reduce Na^+^ concentration in the cytoplasm, which is elevated by saline-alkali stress, plants have evolved the *NHX* gene family. A total of 8 *NHX*s have been found in *Arabidopsis thaliana* (Meng and Wu, 2018). AtNHX1-4 proteins localize to the tonoplast while AtNHX5 and AtNHX6 proteins localize to the Golgi and trans-Golgi networks. The six AtNHXs proteins play an important role in the transport Na^+^ from the cytoplasm to the vacuole and the regulation of pH and K^+^ concentration. The SOS1/AtNHX7 protein localized to the plasma membrane confers saline-alkali tolerance to plants through the SOS (salt overly sensitive) pathway, which excretes Na^+^ from cells. While SOS1B/AtNHX8 was speculated to mediate Li^+^ tolerance in *A. thaliana*, Santander et al. (2021) found that inoculation with *F. mosseae* and *Claroideoglomus lamellosum* increase the expression of *LsaNHX2*, *LsaNHX4*, *LsaNHX6* and *LsaNHX8*, thereby improving Na^+^ tolerance in *Lactuca sativa*. However, the effects of AM fungi on host genes are different under different conditions so that more research is needed.

Therefore, in order to explore the effect of AM fungi on the saline-alkali tolerance of *P. simonii*×*P. nigra*, this pot simulation experiment was carried out. In this study, by measuring the biomass, photosynthesis, gas exchange, ion concentration, the expression of *PxNHX*s and other parameters of mycorrhized poplar growing in a saline-alkali environment, the mechanism of action of AM fungi on *P. simonii*×*P. nigra*’s saline-alkali tolerance was preliminarily clarified. Combined with previous studies, we made the following hypothesis: (1) inoculation with AM fungi improved the saline-alkali tolerance of *P. simonii*×*P. nigra*; (2) inoculation with AM fungi changed the expression pattern of *NHX* gene family in *P. simonii*×*P. nigra*, directly or indirectly affected the photosynthesis, ion absorption and other life activities of *P. simonii*×*P. nigra*, thus enhancing the saline-alkali tolerance of *P. simonii*×*P. nigra*.

## 2 Materials and methods

### 2.1 Plant material

*Populus simonii*×*P. nigra* cuttings were purchased from Lindian County, Daqing City, Heilongjiang Province, China. The cuttings were propagated by the plant factory of Northwest A&F University to obtain clone seedlings.

### 2.2 Fungal material

*Funneliformis mosseae* (BGC XJ01A) was purchased from the Institute of Plant Nutrition, Resources and Environment, Beijing Academy of Agriculture and Forestry Sciences. Propagation of *F. mosseae* using tobacco plants as a host. The tested inoculants included spores (the spore density was approximately 81 per gram of inoculant), mycelia and root fragments.

### 2.3 Growth substrate

Surface soil (5-20 cm) was obtained from the garden of Northwest Agriculture and Forestry University. The soil was passed through a 2 mm sieve and sterilized by moist heat at 121°C for 2 h twice. The river sand was passed through a 2 mm sieve, then washed, and sterilized by dry heat at 170°C for 12 h. The sterile soil and sterile sand were mixed uniformly at the 1:2 (V/V) ratio. The physicochemical properties of the soil substrate were pH 7.6 (soil and water ratio was 1:5), available N 25.77 mg·kg^-1^, available P 12.30 mg·kg^-1^, available K^+^ 112.67  mg·kg^-1^, Ca^2+^ 78.51 mg·kg^-1^, water soluble Na^+^ 203.54 mg·kg^-1^.

### 2.4 Experimental design

The experiment consisted of a randomized complete block design with two factors: inoculation with *F. mosseae* (AM) and no inoculation with *F. mosseae* (NM); saline-alkali stress (S) and no saline-alkali stress (N). There were 18 replicates for each treatment with a total of 72 pots.

100 mmol·L^-1^ Na^+^, pH 9.5 saline-alkali stress solution (the stress concentration was selected based on the data of the seven-year-old *P. simonii*×*P. nigra* rhizosphere soil sample and the data of the pre-test of potted plants in the greenhouse) was made by mixing 50 mmol·L^-1^ Na_2_CO_3_ solution and 100 mmol·L^-1^ NaHCO_3_ solution according to the volume ratio of 4:5. Each pot (7 cm × 7 cm × 5 cm) was preloaded with 230 g growth substrate. The growth substrate in 36 pots (group S) was pretreated: each pot was filled with 35 mL of saline-alkali stress solution (the maximum water content of 250 g soil matrix was about 70 mL), then after drying at 25°C, 35 mL of saline-alkali stress solution was added again. The control group without saline-alkali stress (group N) was treated by the same amount of distilled water using the same method.

Subsequently, 18 pots of each of the two groups (S and N) were selected and added with 20 g *F. mosseae* inoculum. The control group was added with 20 g of *F. mosseae* inoculum after moist heat sterilization.

One seedling was planted in one pot. Plants were cultivated in the greenhouse of the Forestry College of Northwest A&F University. The greenhouse’ room temperature was 25-28°C, light intensity was 3000 lux, photoperiod was 16/8 h, and humidity was 50%-70%. Each pot was weighed and rehydrated daily to keep the soil well moist. Each Pot was watered weekly with 25 mL of Hogland’s nutrient solution (Johnson et al., 1957) containing 1/10 Pi. After 8 weeks of culture, relevant data were measured and samples were collected.

### 2.5 Determination of chlorophyll fluorescence parameters and gas exchange parameters

According to the method of Wu et al. (2016), the third fully expanded leaf was selected for 30 min shading treatment. Then PAM Fluorometer (Mini-imaging-PAM, Walz, Germany) was used to measure the initial fluorescence value (Fo) and maximum fluorescence value (Fm) of leaves under saturation pulse; the minimum fluorescence value (Fo’), maximum fluorescence value (Fm’), and steady-state fluorescence value (Fs) after light adaptation. The maximum quantum yield of PSII (Fv/Fm, Fv/Fm = (Fm – Fo)/Fm), photosynthetic electron transport rate (ETR, ETR = (Fm’ – Fs)/Fm’ × Fm), non-photochemical quenching (qN, qN = (Fm − Fm’)/Fm’), photochemical quenching (qP, qP = (Fm’ – Fs)/(Fm’ − Fo’)), non-photochemical quenching coefficient according to the following formulas (NPQ, NPQ = (Fm – Fm’)/(Fm’)) and PSII actual photon quantum efficiency (ФPSII, ФPSII = (Fm’ – Fs)/Fm’) were calculated by the instrument.

According to the method of Huang et al. (2010), during the period from 8:30 am to 11:30 am, the third fully expanded leaf of each plant was selected for measuring net photosynthetic rate (*P_n_*), stomatal conductance (*g_s_*), intercellular CO_2_ concentration (*C_i_*), and transpiration rate (*E*) by using li-6400 portable open flow gas-exchange system (Li-Cor Inc., Lincoln, NE, United States). The measurement conditions: photo-synthetically active irradiation, 1000 µmol·m^-2^·s^-1^; temperature, 25°C; relative humidity, 60%; and CO_2_ concentration, 400 µmol·mol^-1^.

### 2.6 Plant height, fresh weight measurement and sample collection

After 8 weeks of culturing in the greenhouse, the height of each plant was measured using a ruler (cm). Plant samples were collected in two groups, the aboveground part and the underground part. Each 6 plants were pooled into one replicate, with three replicates per treatment. The fresh weight of the aboveground and underground parts were weighed by using an electronic analytical balance (FA2004A, Shanghai Jingda). The aboveground samples were divided into two parts with one of which immediately frozen in liquid nitrogen, then ground into powder and stored in a -80°C refrigerator and the other of which dried at 75°C to a constant weight. The underground part was divided into three parts, two of which followed the same procedure as aboveground samples and the third part was deposited in the FAA fixative to determine *F. mosseae* infection.

### 2.7 Tissue water content and relative water content of aboveground part determination

Tissue water content (TWC) and relative water content (RWC) in aboveground parts were determined according to the method of Kovar et al. (2019). We obtained the same part of the aboveground part from each sample, and weighed the fresh weight (FW). The samples were immersed in sterile distilled water for 48 hours, and the saturated fresh weight (WT) was weighed. Then the samples were dried at 75°C until constant weight, and the dry weight (DW) was weighed. Finally, the TWC ((FW – DW)/FW × 100%) and RWC ((FW – DF)/(WT – DW) × 100%) were calculated.

### 2.8 AM fungal infection rate detection

Roots were stained with trypan blue (Phillips and Hayman, 1970), and then mycorrhizal colonization was determined using the gridline intersection method (Giovannetti and Mosse, 1980).

### 2.9 Growth substrate pH determination

Referring to the method of Wang et al. (2021), weighed 10 g of air-dried soil samples that have passed through a 1 mm sieve, and measure the soil pH according to the water-soil ratio of 5:1 (W/V).

### 2.10 Plant Na^+^, K^+^, Ca^2+^ determination

After drying, the aboveground and underground samples were ground into powder, dried at 80°C to constant weight, and passed through a 20 μm sieve (Wu et al., 2016). Weighed 0.05 g of plant samples, with reference to the method of Zhou (2022), determine the concentration of Na^+^ and K^+^ in plants. Weighed 0.05 g of plant sample, with reference to the method of Porcel et al. (2016), determine the concentration of Ca^2+^ in plants.

### 2.11 Analysis of the *NHX* gene families of *P. simonii*×*P. nigra*

*P. simonii×P. nigra* genome data was obtained from http://www.wangsui.net.cn/resource/temp/Pxiaohei_DH_1_21_genome_v4.6.tar.gz (Zhou et al., 2019). The NHX protein sequences of *Arabidopsis thaliana* (https://phytozome-next.jgi.doe.gov/info/Athaliana_TAIR10) were used as the input sequences for the BLAST software (2.12.0+) to search for *P. simonii*×*P. nigra NHX* genes (*PxNHX*s). The protein sequences of candidate gene were input into HMMER software (3.3.2) and aligned with the PFAM database (https://ftp.ebi.ac.uk/pub/databases/Pfam/releases/Pfam35.0/Pfam-A.hmm.gz) to further verify their domains.

Protein physicochemical properties of *PxNHX*s were analyzed using ProtParam (https://web.expasy.org/protparam/). Subcellular localization predictions of *PxNHX*s were performed using Plant-mPLoc (http://www.csbio.sjtu.edu.cn/bioinf/plant-multi/#). Transmembrane domain predictions of *PxNHX*s were performed using TMHMM 2.0 (https://services.healthtech.dtu.dk/service.php?TMHMM-2.0). Signal peptide predictions for *PxNHXs* were performed using SignalP 5.0 (https://services.healthtech.dtu.dk/service.php?SignalP-5.0).

Phylogenetic tree of the *NHX* gene families of *P. simonii*×*P. nigra*, *A. thaliana*, *Zea mays* (Zorb et al., 2005), and *P. trichocarpa* (Tian et al., 2017) were constructed using MEGA X using the adjacency method (NJ) and 1000 bootstrap-up replicates. The phylogenetic tree was visualized by iTOL (https://itol.embl.de/).

### 2.12 Quantitative analysis of *PxNHX*s

Total RNA was extracted from these samples using the E.Z.N.A Plant RNA Kit R6827-01 (Omega Bio-Tek, Norcross, GA, USA) following the manufactory instructions strictly. RNA was reversely transcribed to cDNA using the FastQuant RT Kit KR106 (TIANGEN, Beijing, China) according to the manufactory instructions. By using NCBI Primer-BLAST (https://www.ncbi.nlm.nih.gov/tools/primer-blast/index.cgi?LINK_LOC=BlastHome), the *PxNHX*s’ quantitative primers was designed (**Supplementary Table 1**). Two housekeeping genes (NCBI accession: BU875027, GQ253565.1) were served as the reference genes (Dong et al., 2021). The qRT-PCR reaction was conducted by the CF96X real-time PCR system (Bio-Rad, Hercules, CA, USA). Each reaction mixture was 10 µL containing 1 µL diluted cDNA template, 0.5 µL forward and reverse primers (10 µmol·L^-1^), 5 µL ChamQ SYBR qPCR Master Mix (Vazyme, Nanjing, China), and 3 µL sterilized ddH_2_O. The two-step qRT-PCR was run as follows: 30 s denaturation at 95°C, 40 cycles of denaturation at 95°C for 10 s, annealing at the annealing temperature for 10 s, extension at 72°C for 20 s, followed by heating from 65 to 95°C at a rate of 0.5°C every 5 s. All samples were amplified in duplicates from the same RNA preparation and the mean value was calculated. Meanwhile, we averaged three amplified samples from each RNA and the relative expression of each target gene was calculated according to the 2^−ΔΔCT^ protocol (Livak and Schmittgen, 2001).

### 2.13 Statistical analysis

All experiments were repeated at least three times. All results were expressed as the mean ± standard error (SE) in tables and figures. One-way and two-way analysis of variance (ANOVA) and Tukey’s tests using SPSS software (Version 26.0, SPSS Inc., Chicago, IL, USA) were used to evaluate significant differences across all parameters.

## 3 Results

### 3.1 Effects of saline-alkali stress on AM fungal infection rate

**Figure 1:**
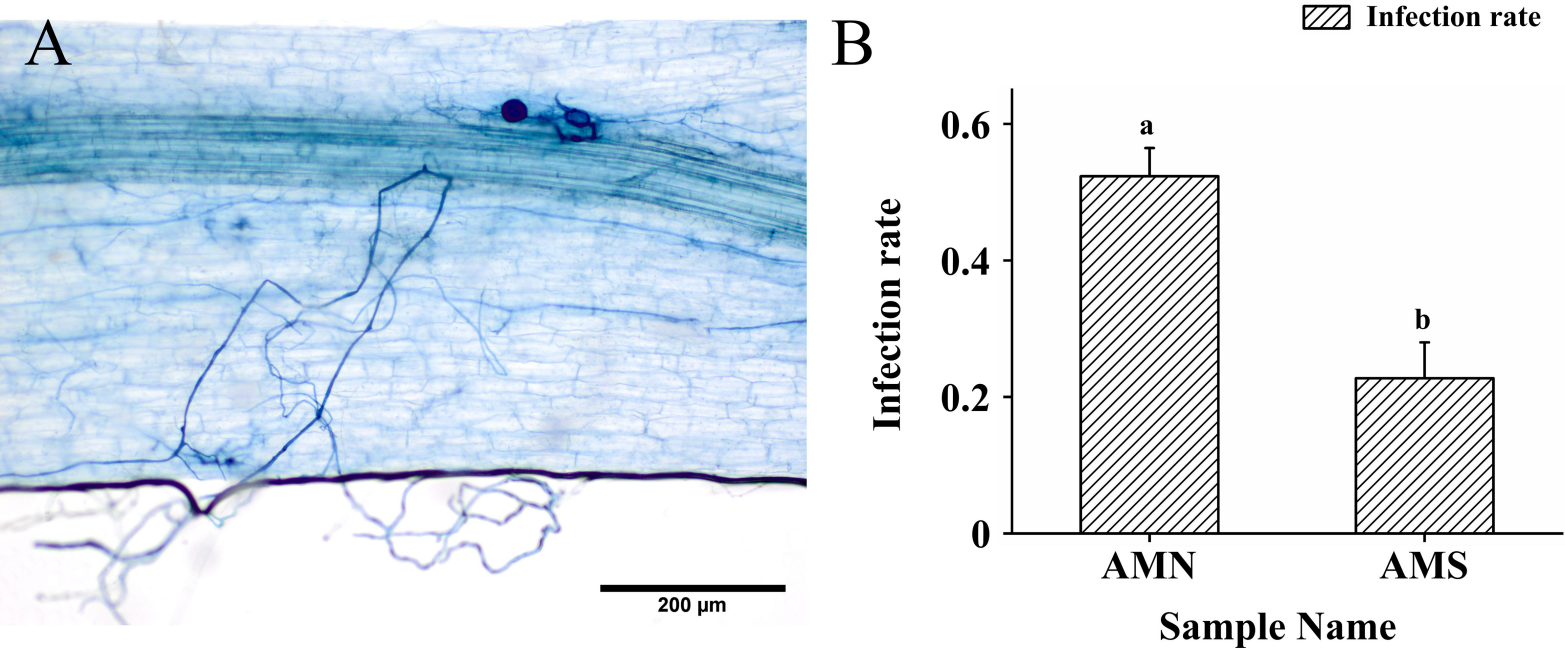
AM fungal infection photos and changes of AM fungal infection rate under saline-alkali stress. **(A)** AM fungal infection photos; **(B)** Changes of AM fungal infection rate under saline-alkali stress. AMN means inoculation with *F. mosseae* without saline-alkali stress; AMS means inoculation with *F. mosseae* and application of saline-alkali stress. Bars indicate mean ± standard error (n = 3). Different lowercase letters indicate significant differences between the means by Tukey’s test (*P* < 0.05).

After 8 weeks, the roots of *P. simonii*×*P. nigra* established a symbiotic relationship with *F*. *mosseae* (**Figure 1A**). Under saline-alkali stress, the infection rate was significantly decreased by 56.56% (**Figure 1B**), which indicated that the infection activity of AM fungi was inhibited by saline-alkali stress.

### 3.2 Effects of AM fungal inoculation and saline-alkali stress on soil pH

**Figure 2:**
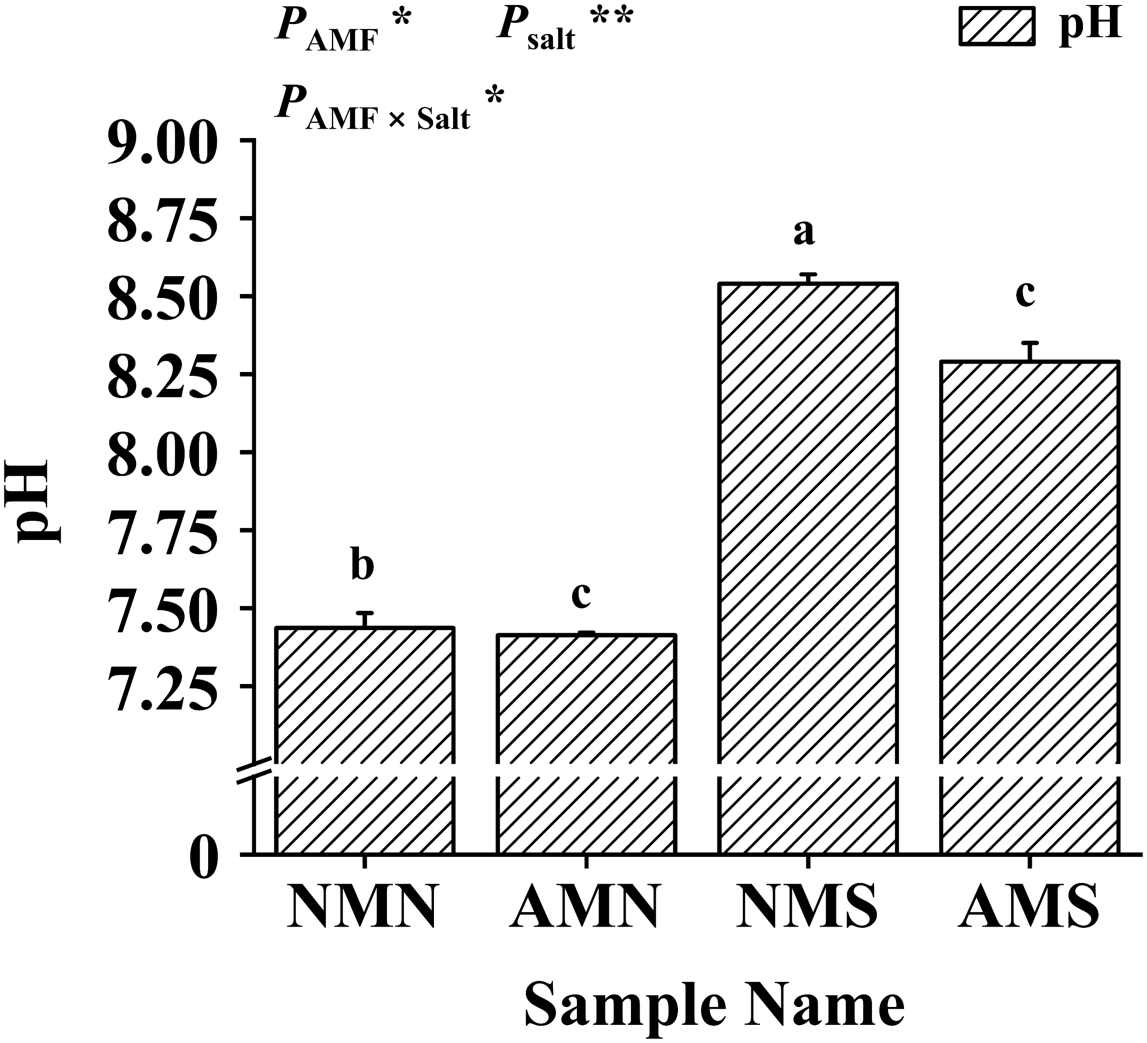
Effects of AM fungal inoculation and saline-alkali stress on soil pH. NMN means no *F. mosseae* inoculation and no saline-alkali stress; AMN means inoculation with *F. mosseae* without saline-alkali stress; NMS means application of saline-alkali stress without *F. mosseae* inoculation; AMS means inoculation with *F. mosseae* and application of saline-alkali stress. Bars indicate mean ± standard error (n = 3). Different lowercase letters indicate significant differences between the means by Tukey’s test (*P* < 0.05). Significant effect of two-way ANOVA analysis: “*” indicates *P* < 0.05, “**” indicates *P* < 0.01.

As shown in **Figure 2**, saline-alkali treatment significantly increased soil pH while *F*. *mosseae* inoculation decreased soil pH. The soil pH of NMS (i.e., 8.54) was the highest. While the soil pH of AMS was significantly reduced by 2.93%. Two-way ANOVA showed that soil pH was significantly affected by *F*. *mosseae* inoculation and saline-alkali stress. The results indicated that AM fungi played an important role in reducing the pH of the rhizosphere soil of *P. simonii*×*P. nigra*.

### 3.3 Effects of AM fungal inoculation and saline-alkali stress on plant height and fresh weight of *P. simonii*×*P. nigra*

**Figure 3:**
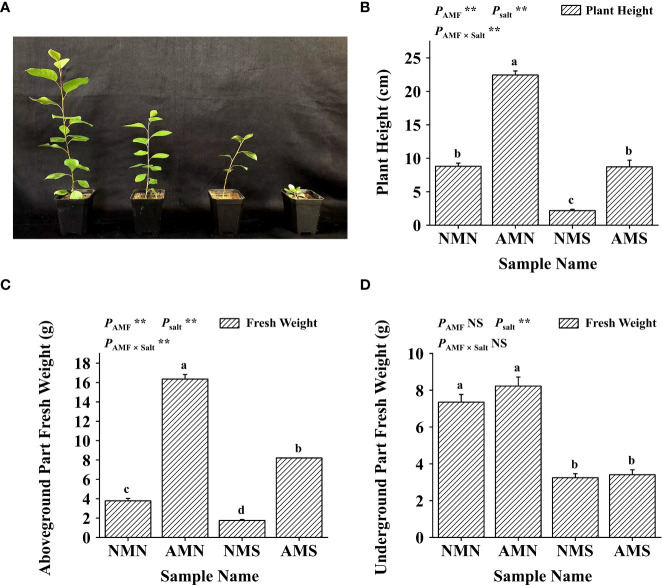
Effects of AM fungal inoculation and saline-alkali stress on plant height and fresh weight of *P. simonii*×*P. nigra*. **(A)** Photos of *P. simonii*×*P. nigra* growth status after saline-alkali stress and AM fungus inoculation for 8 weeks; from left to right, AMN, AMS, NMN and NMS. **(B)** Plant height histogram of *P. simonii*×*P. nigra*, bars indicate mean ± standard error (n = 18). **(C)** The fresh weight histogram of the aboveground part of *P. simonii*×*P. nigra*, bars indicate mean ± standard error (n = 3). **(D)** The fresh weight histogram of the underground part of *P. simonii*×*P. nigra*, bars indicate mean ± standard error (n = 3). NMN means no *F. mosseae* inoculation and no saline-alkali stress; AMN means inoculation with *F. mosseae* without saline-alkali stress; NMS means application of saline-alkali stress without *F. mosseae* inoculation; AMS means inoculation with *F. mosseae* and application of saline-alkali stress. Different lowercase letters indicate significant differences between the means by Tukey’s test (*P* < 0.05). Significant effect of two-way ANOVA analysis: “**” indicates *P* < 0.01; “NS” indicates no interaction (*P* ≥ 0.05).

As shown in **Figure 3**, saline-alkali stress significantly inhibited the growth of *P. simonii*×*P. nigra*. Compared with NMN, the plant height, fresh weight of aboveground part and fresh weight of underground part of NMS decreased by 75.25%, 53.69% and 53.69%, respectively, and AMS of which decreased by 61.16%, 49.80% and 58.52%, respectively compared with AMN. However, under the influence of *F. mosseae* inoculation, the plant height, fresh weight of aboveground part and fresh weight of underground part of AMS were 300.51%, 368.74% and 5.31% higher than those of NMS, respectively. Two-way ANOVA showed that the plant height and fresh weight of the aboveground part of *P. simonii*×*P. nigra* were significantly affected by AM fungal inoculation and saline-alkali treatment, indicating that AM fungi played an important role in promoting the growth of the aboveground part of *P. simonii*×*P. nigra*.

### 3.4 Effects of AM fungal inoculation and saline-alkali stress on water content

The fresh weight of plants was affected by water content, so the tissue water content (TWC) and relative water content (RWC) of the aboveground part was further investigated. As shown in **Table 1**, under the influence of saline-alkali stress, the TWC of AMS and NMS was significantly increased, but RWC was not significantly affected, probably because *P. simonii*×*P. nigra* had adapted to the long-term stressed environment. Inoculation with *F. mosseae* could significantly increase the TWC and RWC of the aboveground parts. Compared with NMS, the TWC of AMS was significantly increased by 23.74%, and the RWC was increased by 11.70%, indicating that AM fungi play an important role in promoting plant water uptake.

**Table 1:** Tissue water content and relative water content of aboveground part of *P. simonii*×*P. nigra* under different treatments.

Sample	NMN	AMN	NMS	AMS	*P*_AMF_	*P*_saline-alkali_	*P*_saline-alkali*AMF_
TWC (%)	61.93 ± 0.95 c	73.17 ± 0.56 b	64.54 ± 1.31 c	79.86 ± 0.36 a	**	**	*
RWC (%)	85.18 ± 0.53 b	94.79 ± 0.15 a	85.30 ± 0.73 b	95.28 ± 0.23 a	**	NS	NS

TWC means tissue water content; RWC means relative water content. NMN means no F. mosseae inoculation and no saline-alkali stress; AMN means inoculation with F. mosseae without saline-alkali stress; NMS means application of saline-alkali stress without F. mosseae inoculation;AMS means inoculation with F. mosseae and application of saline-alkali stress. Data expressed as mean ± standard error (n = 3). Different lowercase letters indicate significant differences between the means by Tukey’s test (P < 0.05). Significant effect of two-way ANOVA analysis: “*” indicates P < 0.05, “**” indicates P < 0.01; “NS” indicates no interaction (P ≥ 0.05).

### 3.5 Effects of AM fungal inoculation and saline-alkali stress on gas exchange parameters

**Figure 4:**
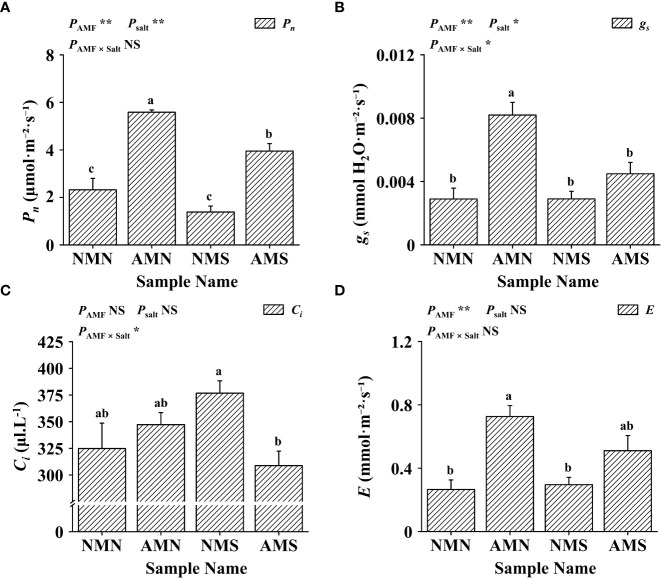
Effects of AM fungal inoculation and saline-alkali stress on gas exchange parameters. **(A)** Effects of AM fungal inoculation and saline-alkali stress on *P_n_* value; **(B)** Effects of AM fungal inoculation and saline-alkali stress on *g_s_* value; **(C)** Effects of AM fungal inoculation and saline-alkali stress on *C_i_* value; **(D)** Effects of AM fungal inoculation and saline-alkali stress on *E* value. NMN means no *F. mosseae* inoculation and no saline-alkali stress; AMN means inoculation with *F. mosseae* without saline-alkali stress; NMS means application of saline-alkali stress without *F. mosseae* inoculation; AMS means inoculation with *F. mosseae* and application of saline-alkali stress. *P_n_* means net photosynthetic rate, *g_s_* means stomatal conductance, *C_i_* means intercellular CO_2_ concentration, and *E* means transpiration rate. Bars indicate mean ± standard error (n = 5). Different lowercase letters indicate significant differences between the means by Tukey’s test (*P* < 0.05). Significant effect of two-way ANOVA analysis: “*” indicates *P* < 0.05, “**” indicates *P* < 0.01; “NS” indicates no interaction (*P* ≥ 0.05).

Plants synthesize organic matter through photosynthesis for maintaining a symbiotic relationship with mycorrhizae and for their own growth. As shown in **Figure 4**, *F. mosseae* inoculation increased the *P_n_* (net photosynthetic rate, 140.57%), *g_s_* (stomatal conductance, 183.33%), *C_i_* (intercellular CO_2_ concentration, 6.88%) and *E* (transpiration rate, 173.11%) of *P. simonii*×*P. nigra* without salt-alkali stress. The application of saline-alkali stress resulted in a decrease in the *P_n_* of NMS (40.25%) and a significant increase in the *C_i_* (15.97%). For AMS, although saline-alkali stress led to a significant decrease in *P_n_* (29.28%), *g_s_* (45.27%), *C_i_* (11.05%) and *E* (29.69%) (compared with AMN), the *P_n_* (70.13%), *g_s_* (55.07%) and *E* (92.03%) were still higher than those of NMN. Two-way ANOVA analysis showed that *g_s_* and *C_i_* were affected between saline-alkali stress and AM fungi interaction, indicating that AM fungi may promote the growth of *P. simonii*×*P. nigra* by regulating these two pathways under saline-alkali stress.

### 3.6 Effects of AM fungal inoculation and saline-alkali stress on chlorophyll fluorescence

**Figure 5:**
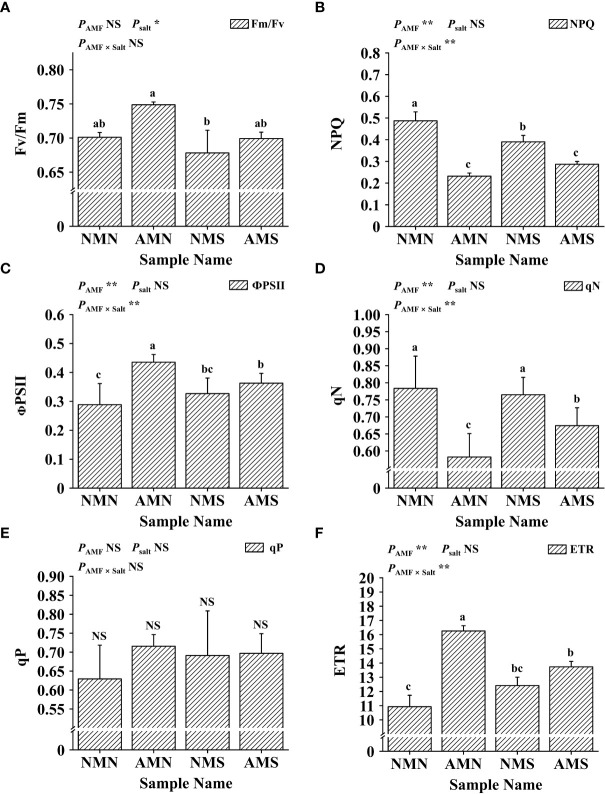
Effects of AM fungal inoculation and saline-alkali stress on chlorophyll fluorescence. **(A)** Effects of AM fungal inoculation and saline-alkali stress on Fv/Fm value; **(B)** Effects of AM fungal inoculation and saline-alkali stress on NPQ value; **(C)** Effects of AM fungal inoculation and saline-alkali stress on ФPSII value; **(D)** Effects of AM fungal inoculation and saline-alkali stress on qN value; **(E)** Effects of AM fungal inoculation and saline-alkali stress on qP value; **(F)** Effects of AM fungal inoculation and saline-alkali stress on ETR value. Fv/Fm means maximal photosystem II quantum yield; NPQ means non-photochemical quenching; ФPSII means the efficiency of photosystem II; qN means non-photochemical quenching coefficient; qP means photochemical quenching coefficient; ETR means relative electron transfer rate. NMN means no *F. mosseae* inoculation and no saline-alkali stress; AMN means inoculation with *F. mosseae* without saline-alkali stress; NMS means application of saline-alkali stress without *F. mosseae* inoculation; AMS means inoculation with *F. mosseae* and application of saline-alkali stress. Bars indicate mean ± standard error (n = 5). Different lowercase letters indicate significant differences between the means by Tukey’s test (*P* < 0.05). Significant effect of two-way ANOVA analysis: “*” indicates *P* < 0.05, “**” indicates *P* < 0.01; “NS” indicates no interaction (*P* ≥ 0.05).

As shown in **Figure 5**, the effects of saline-alkali stress and AM fungal inoculation on the chlorophyll fluorescence parameters of *P. simonii*×*P. nigra* were different. Saline-alkali stress significantly reduced the Fv/Fm value regardless of whether *F. mosseae* was inoculated or not. However, the Fv/Fm value of AMS was still significantly higher than that of NMS (3.10%), which was similar to that of NMN. For NPQ, ФPSII, qN, qP and ETR, the inoculation with *F. mosseae* under saline-alkali stress had opposite effects on these parameters. Compared with NMN, NPQ and qN of NMS decreased by 19.90% and 2.39%, respectively while ФPSII, qP and ETR increased by 13.28%, 9.82% and 13.61%, respectively. Although inoculation with *F. mosseae* increased the values of ФPSII, qP and ETR (compared with NMN), under the influence of saline-alkali stress, compared with AMN, the ФPSII, qP and ETR values of AMS decreased by 16.63%, 2.61% and 15.50%, respectively, and at the same time, NPQ and qN increased by 23.67% and 13.64%, respectively. However, the ФPSII (11.08%), qP (0.84%) and ETR (10.64%) of AMS were still higher than those of NMS. Two-way ANOVA analysis showed that AM fungal inoculation and saline-alkali stress significantly affected NPQ, ФPSII, qN and ETR.

### 3.7 Effects of AM fungus inoculation and saline-alkali stress on Na^+^, K^+^ and Ca^2+^ concentrations of *P. simonii*×*P. nigra*

As shown in **Table 2**, the saline-alkali treatment caused an increase in the Na^+^, K^+^ and Ca^2+^ concentrations of *P. simonii*×*P. nigra*, while the Na^+^, K^+^ and Ca^2+^ concentrations were further increased after inoculation with AM fungi. Compared with NMS, the Na^+^, K^+^ and Ca^2+^ concentrations in the aboveground part of AMS were increased by 444.92%, 63.14% and 62.31%, respectively while those of the underground part were increased by 106.28%, 8.53% and 0.93%, respectively. Two-way ANOVA showed that the Na^+^, K^+^ and Ca^2+^ concentrations in the aboveground and underground parts of *P. simonii*×*P. nigra* were significantly affected by AM fungal inoculation and saline-alkali treatment. It suggested that AM fungal inoculation in saline-alkali environment played an important role in promoting plant element uptake.

**Table 2:** Na^+^, K^+^ and Ca^2+^ concentrations in aboveground and underground parts of *P. simonii*×*P. nigra*.

Sample	Na (g·kg^-1^)		K (g·kg^-1^)		Ca (g·kg^-1^)	
	aboveground	underground	aboveground	underground	aboveground	underground
NMN	0.11 ± 0.029 c	1.22 ± 0.29 b	23.16 ± 1.40 b	12.53 ± 0.52 b	3.92 ± 0.29 b	2.18 ± 0.089 b
AMN	0.62 ± 0.074 bc	2.64 ± 0.65 b	31.32 ± 3.56 b	20.34 ± 1.29 a	4.62 ± 0.35 b	3.14 ± 0.20 a
NMS	1.18 ± 0.21 b	3.66 ± 0.25 b	27.29 ± 1.06 b	12.31 ± 0.14 b	4.67 ± 0.14 b	2.16 ± 0.042 b
AMS	6.43 ± 0.29 a	7.55 ± 0.95 a	44.52 ± 1.090 a	13.36 ± 0.24 b	7.58 ± 0.20 a	2.18 ± 0.11 b
*P*_saline-alkali_	**	**	**	**	**	**
*P*_AMF_	**	NS	NS	**	**	**
*P*_saline-alkali*AMF_	**	**	**	**	**	**

NMN means no F. mosseae inoculation and no saline-alkali stress; AMN means inoculation with F. mosseae without saline-alkali stress; NMS means application of saline-alkali stress without F. mosseae inoculation;AMS means inoculation with F. mosseae and application of saline-alkali stress. Data expressed as mean ± standard error (n = 3). Different lowercase letters indicate significant differences between the means by Tukey’s test (P < 0.05). Significant effect of two-way ANOVA analysis: “**” indicates P < 0.01; “NS” indicates no interaction (P ≥ 0.05).

### 3.8 Identification and analysis of *NHX* gene families of *P. simonii*×*P. nigra*

A total of 8 *NHX* gene family members were identified in the *P. simonii*×*P. nigra* genome (**Supplementary Table 2**). The protein length of the eight *PxNHX*s ranged from 318 (Px_DH25939) to 1147 (Px_DH21638) amino acids; the molecular weight of the protein ranged from 35.13 kDa (Px_DH25939) to 127.02kDa (Px_DH21638); the isoelectric point ranged from 5.48 (Px_DH25989) to 8.85 (Px_DH28184); the number of transmembrane domains ranged from 7 (Px_DH25939) to 12 (Px_DH18325 and Px_DH21638). Subcellular localization showed that six *PxNHX*s were localized to vacuole and other two *PxNHX*s were localized to the cell membrane.

**Figure 6:**
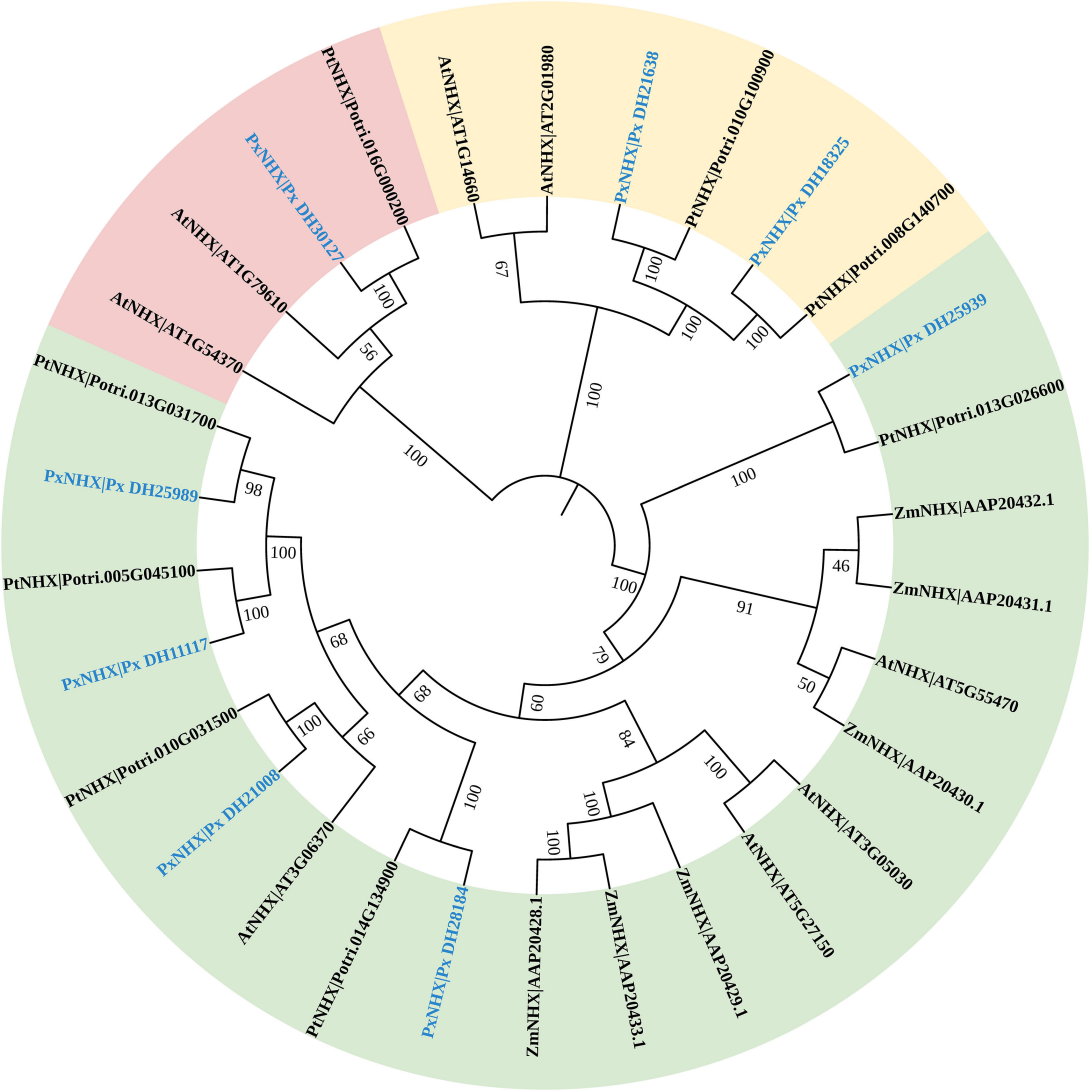
The evolutionary tree of the *NHX* gene family of *P. simonii*×*P. nigra*, *Arabidopsis thaliana*, *P. trichocarpa* and *Zea mays*.

To further clarify the corresponding gene functions and evolutionary relationships of *PxNHX*s, a phylogenetic tree was constructed (**Figure 6**). The eight *PxNHX*s were divided into three clades: Px_DH25939, Px_DH28184, Px_DH21008, Px_DH25989 and Px_DH11117 were divided into the same clades, which were similar to *AtNHX2* in function; Px_DH30127 was divided into one branch, which was functionally similar to *AtNHX6*; Px_DH21638 and Px_DH18325 were divided into the same clade and were functionally similar to *AtNHX7*.

### 3.9 Effects of AM fungal inoculation and saline-alkali stress on the expression of *PxNHX*s

**Figure 7:**
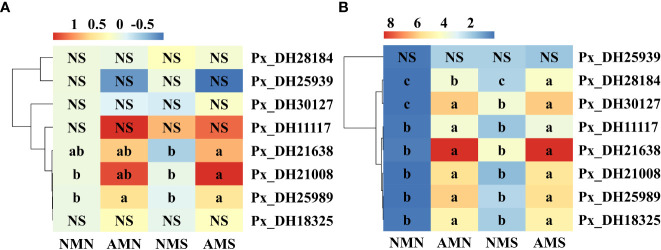
Expression thermogram of *NHX* gene family in *P. simonii*×*P. nigra*. **(A)** Expression thermogram of *NHX* gene family in aboveground part of *P. simonii*×*P. nigra*. **(B)** Expression thermogram of *NHX* gene family in underground part of *P. simonii*×*P. nigra*. NMN means no *F. mosseae* inoculation and no saline-alkali stress; AMN means inoculation with *F. mosseae* without saline-alkali stress; NMS means application of saline-alkali stress without *F. mosseae* inoculation;AMS means inoculation with *F. mosseae* and application of saline-alkali stress. The colour scale represents the log2 value of the gene expression (n = 3). From red to blue, the expression level was from high to low. Different lowercase letters indicate significant differences between the means by Tukey’s test (*P* < 0.05). “NS” indicates no interaction (*P* ≥ 0.05). The results of two-way ANOVA for each gene are presented in the **Supplementary Table 3**.

Through qPCR analysis of *NHX* gene family, the effects of AM fungus inoculation on saline-alkali tolerance of *P. simonii*×*P. nigra* were explored from the microscopic level. In the aboveground part (**Figure 7A**), saline-alkali stress led to the down-regulated expression of Px_DH21008, Px_DH25989, Px_DH30127, Px_DH21638, and Px_DH18325 in NMS to varying degrees (2.45%-25.36%). Under saline-alkali stress, inoculation with *F. mosseae* up-regulated the expression of *PxNHX*s except Px_DH25939. Among them, Px_DH21008, Px_DH25989, Px_DH11117 and Px_DH21638 were significantly affected by *F. mosseae* inoculation, and their expressions were up-regulated. Compared with NMS, their expression levels in AMS were increased by 182.95%, 57.24%, 27.34%, and 143.56%, respectively. In the underground part (**Figure 7B**), *PxNHX*s’ expressions were more active. Under the saline-alkali stress, these genes were up-regulated by 200.99% (Px_DH21008)-1562.91% (Px_DH21638) in NMS. Inoculation with *F. mosseae* also induced further up-regulation of these genes. In AMS, the expression of these genes increased by 254.16% (Px_DH25939)-34762.16% (Px_DH21638) compared with NMN, indicating that AM fungi endow plants with higher saline-alkali tolerance by inducing the up-regulated expression of these genes. One-way ANOVA of the underground part showed that saline-alkali stress mainly affected the expression of Px_DH28184 and Px_DH30127. While AM ​​fungi significantly affected the expression of *PxNHX*s except Px_DH25939. Two-way ANOVA indicated that the expression of Px_DH11117 and Px_DH30127 in the underground part were affected by the interaction of AM fungal inoculation and saline-alkali treatment.

### 3.10 Correlation analysis of *PxNHX*s induced by AM fungi and growth state of *P. simonii*×*P. nigra*

**Figure 8:**
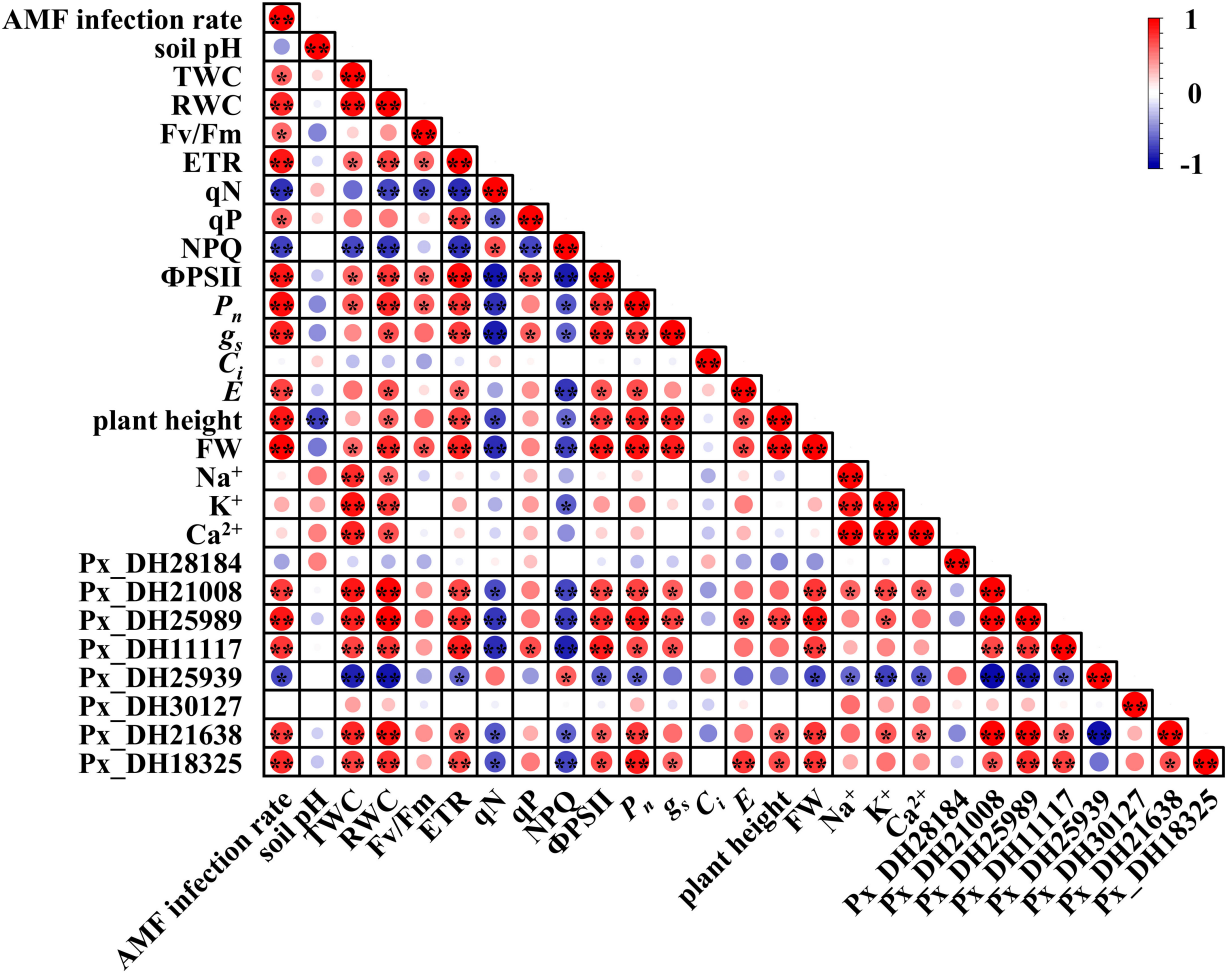
Thermogram of Pearson’s correlation between AM fungal infection and different parameters in aboveground part. TWC, tissue water content; RWC, relative water content; Fv/Fm, the maximum quantum yield of PSII; ETR, photosynthetic electron transport rate, qN, non-photochemical quenching, qP, photochemical quenching, NPQ, non-photochemical quenching coefficient according to the following formulas; ФPSII, PSII actual photon quantum efficiency; *P_n_*, net photosynthetic rate; *g_s_*, stomatal conductance; *C_i_*, intercellular CO_2_ concentration; *E*, transpiration rate; FW, fresh weight. Each circle indicates the Pearson’s correlation coefficient of a pair of parameters. “*” indicates *P* < 0.05, “**” indicates *P* < 0.01.

In order to explore the potential relationship between *PxNHX*s induced by AM fungi and the growth status of *P. simonii*×*P. nigra*, Pearson’s correlation analysis was performed on the above parameters. In aboveground parts (**Figure 8**), inoculation with AM fungi was significantly correlated with the expression of Px_DH21008, Px_DH25989, Px_DH11117, Px_DH25939, Px_DH21638, Px_DH18325. The expression of these genes significantly affected the physiological activities (TWC, RWC, plant weight and FW), photosynthesis (ETR, qN, qP, NPQ and ФPSII), gas exchange (*P_n_* 、*g_s_* and *E*) and ion concentration (Na^+^, K^+^ and Ca^2+^) of the aboveground parts, suggesting that AM fungi could affect the growth status of *P. simonii*×*P. nigra* by affecting the expression of *PxNHX*s in the aboveground parts.

**Figure 9:**
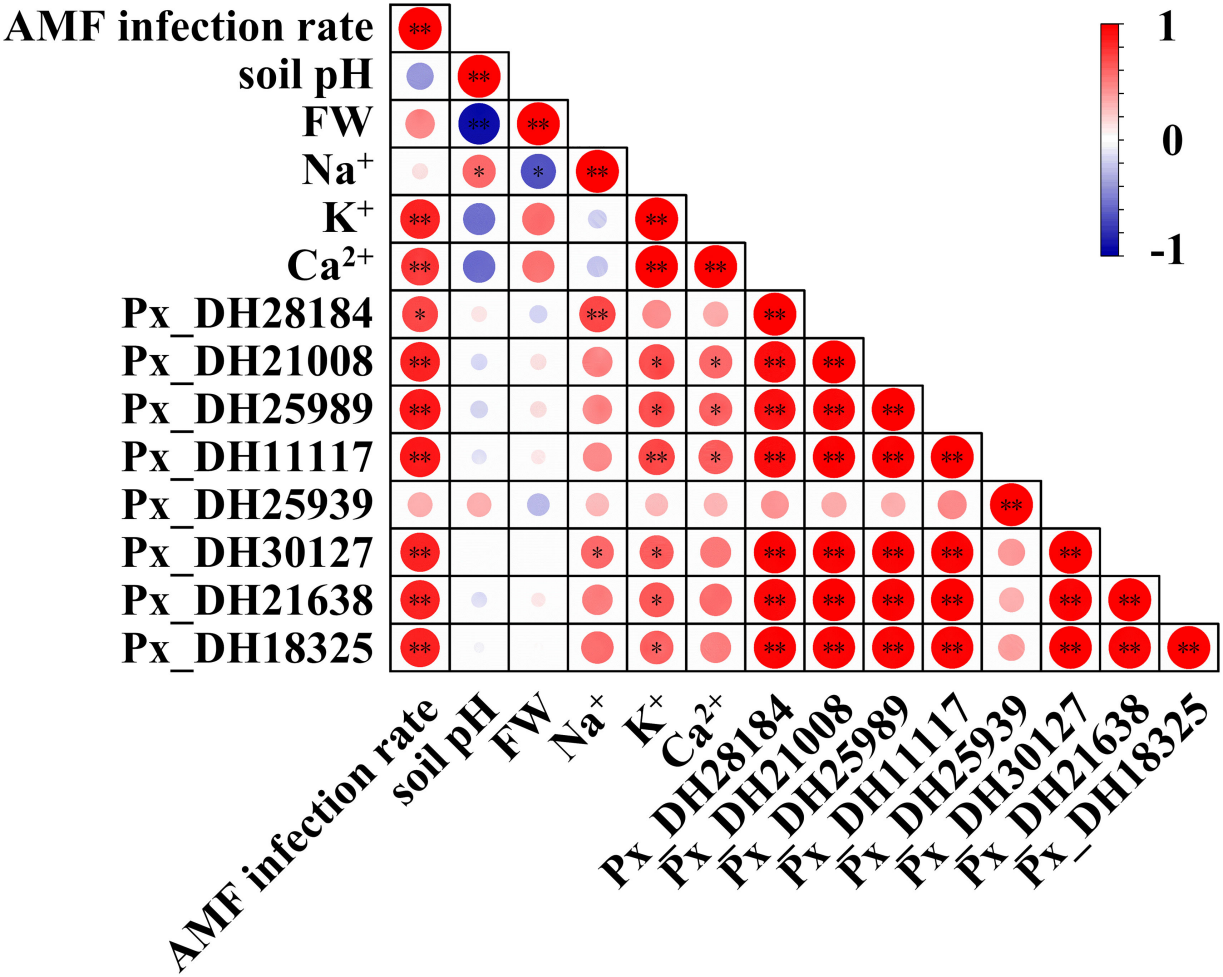
Thermogram of Pearson’s correlation between AM fungal infection and different parameters in underground part. FW, fresh weight. Each circle indicates the Pearson’s correlation coefficient of a pair of parameters. “*” indicates *P* < 0.05, “**” indicates *P* < 0.01.

In underground parts (**Figure 9**), inoculation with AM fungi was significantly correlated with the *PxNHX*s excepted Px_DH25939. At the same time, these genes were also related to the Na^+^, K^+^, Ca^2+^ concentrations in the underground part of *P. simonii*×*P. nigra*. Although AM fungal inoculation had no direct and significant correlation with root Na^+^ concentration and rhizosphere soil pH, the Px_DH28184, Px_DH30127 were significantly correlated with underground Na^+^ concentration, while Na^+^ concentration was significantly correlated with underground FW and rhizosphere soil pH. This result implies that AM fungi can alleviate saline-alkali stress by regulating the expression of these two genes to regulate root growth and reduce soil pH.

## 4 Discussion

Saline-alkali stress seriously endangers the normal growth of plants (Yu et al., 2021). To mitigate this effect, plants take a variety of methods (Fang et al., 2021). Some plants can tolerate saline-alkali environments better by establishing a symbiotic relationship with AM fungi in the rhizosphere (Xun et al., 2015; Zu et al., 2018). Our results showed that the inoculation with *F. mosseae* improved the saline-alkali tolerance of *P. simonii*×*P. nigra*. At the same time, the effects of *F. mosseae* on the saline-alkali tolerance of *P. simonii*×*P. nigra* was manifold.

Under saline-alkali stress, the activities of plants and microorganisms could be inhibited (Arab et al., 2018; Ma et al., 2022a). Previous studies have shown that the saline-alkali tolerance of plants was positively correlated with biomass (Chen et al., 2017). In our study, although the infection activity of *F. mosseae* was inhibited by saline-alkali stress, the establishment of the symbiotic relationship between *F. mosseae* and *P. simonii*×*P. nigra* still promoted the increase of plant height and fresh weight of AMS aboveground parts, indicating that mycorrhizated *P. simonii*×*P. nigra* had higher saline-alkali tolerance than non-mycorrhizated *P. simonii*×*P. nigra*.

Plants absorb CO_2_ and water from the outside, and produce organic matter through a series of redox reactions on chloroplasts for plant growth (Khan et al., 2022). The occurrence of saline-alkali stress hinders the normal water absorption of plants and increases the concentration of Na^+^ and reactive oxygen species in cells. At the same time, the physiological drought of the plant is induced, and the plant reduces the evaporation of water by closing the stomata to retain more water for diluting the Na^+^ in the cells, thereby reducing the stomatal conductance (Athar et al., 2015). On the other hand, salt stress reduces photosynthesis by inhibiting photosystem II (PSII) activity and destroying chlorophyll pigments through Na^+^ accumulation. In salt-sensitive sweet sorghum, salt stress significantly reduced its *P_n_*, ФPSII, *g_s_*, and *C_i_*. In contrast, the salt-tolerant sweet sorghum genotypes could still maintain high photosynthetic efficiency (Sui et al., 2015). Therefore, the strength of photosynthetic ability is also one of the important indicators to measure the stress tolerance of plants. Many studies have confirmed that AM fungi can regulate the expression of photosynthesis-related genes by establishing a symbiotic relationship with plants, increasing the photosynthetic activity of host plants, regulating stomatal conductance, and promoting plant growth in stressful environments (Chandrasekaran et al., 2019; Diao et al., 2021). Under stressed conditions, AM fungi transfer the water in the soil gap to the surrounding of the root through the extra-root hyphae, and on the other hand increase the transpiration rate and stomatal conductance of the host to accelerate water absorption (Zhu et al., 2015). Our study also found that the water content, stomatal conductance and transpiration rate of AMS were significantly higher than those of NMS. This shows that after the occurrence of stress, the roots of *P. simonii*×*P. nigra* can absorb more water by relying on *F. mosseae* to alleviate the physiological drought in the body, so that the stomatal opening degree is higher than that of NMS, which is conducive to the progress of photosynthesis. The replenishment of water and carbon dioxide allows photosynthesis to proceed smoothly. Although the *P_n_* of *P. simonii*×*P. nigra* in AMS and NMS decreased significantly, inoculation with *F. mosseae* still increased the *P_n_* value. Therefore, the *P. simonii*×*P. nigra* in AMS can produce more organic matter for its own growth and secrete organic acids to improve the soil environment of the roots, obtain more nutrients, and form a virtuous circle. In salt-tolerant and sensitive genotypes, Fv/Fm and ΦPSII decreased with increasing NaCl concentration, and the decrease was more pronounced in plants sensitive to salt stress (Yang et al., 2019c). Due to salt-induced increase in Fo and significant decrease in Fm, the Fv/Fm and ФPSII values were significantly reduced after salt stress treatment by Wu et al. (2016), resulting in a decrease in the biomass of *P. cathayana*, while inoculation with *Rhizophagus intraradices* could increase the Fm/Fv and ФPSII values. In this study, it was also found that inoculation of *F. mosseae* under saline-alkali stress increased the Fv/Fm, ФPSII and ETR values of *P. simonii*×*P. nigra*. AMS showed higher Fv/Fm and ФPSII values than NMS, indicating that NMS had a more serious disorder in the ФPSII electron transport chain. Studies have shown that the value of ФPSII and ETR is positively correlated with the tolerance of plants to adversity (Jia et al., 2019). Our results further confirmed that *P. simonii*×*P. nigra* exhibited higher saline-alkali tolerance through long-term symbiosis with *F. mosseae* under the same stressed environment.

The increase of soil pH inhibited the growth and activity of roots, resulting in the immobilization of bioavailable nutrients and then reducing the uptake efficiency of these nutrients by plants (Robin et al., 2016; Neina, 2019). Roots can secrete organic acids to reduce soil pH, dissolve carbonate minerals and release related elements for plant’s absorption, and maintain pH stability and ion balance in cells (Fazeli-Nasab et al., 2022). However, due to factors such as weakened root vigor, plants cannot absorb enough nutrients for the synthesis of organic acids in a short period of time, so they cannot reduce soil pH to a suitable growth level in a short period of time. Inoculation with AM fungi accelerates this process, assisting the plant to reduce the pH of the rhizosphere soil in a shorter time. In *Citrus reticulata*, *Glomus epigaeum* lowered soil pH by inducing the secretion of phenolic acids from roots (Zhang et al., 2020a). The pH of the rhizosphere soil was also significantly decreased after inoculation with *F. mosseae* in *P. simonii*×*P. nigra*. This indicates that *F. mosseae* can promote the secretion of organic acids in *P. simonii*×*P. nigra* to improve the soil environment, but which organic acid secretion is affected needs further study. In addition to inducing plants to secrete organic acids, the mycelia will also secrete organic acids for mineral weathering, while absorbing metal cations in the soil and releasing H^+^, thereby reducing the pH of the rhizosphere soil (Luthfiana et al., 2021; Wang et al., 2022a). Our findings also support this view. The correlation analysis of the underground part of *P. simonii*×*P. nigra* showed that the pH of the soil was significantly correlated with the Na^+^ concentration in the underground part. Whether under saline-alkali stress or not, the presence of AM fungi increased Na^+^ concentration in *P. simonii*×*P. nigra* roots and decreased rhizosphere soil pH. Ma et al. (2022b) found that AM fungi mediated the uptake of Na^+^ and the efflux of H^+^ in the root of *Ziziphus jujuba* under salt stress. These results indicated that AM fungi could improve the rhizosphere soil environment and reduce stress damage to the root system by promoting the absorption of Na^+^ and releasing H^+^ to reduce the pH of the rhizosphere.

The increase of single Na^+^ concentration in cells disrupts ion homeostasis, affects enzyme activity and cell membrane stability, and thus affects normal cell growth. Since Na^+^ and K^+^ have similar structures, they compete with each other for adsorption sites and active sites, resulting in reduced K^+^ absorption and inhibition of K^+^-dependent enzymatic activities and metabolic processes (Assaha et al., 2017). At the same time, Na^+^ can also replace Ca^2+^ on the cell membrane, thereby destroying the membrane structure (Lu et al., 2018). Therefore, plants need to absorb more K^+^ and Ca^2+^ from the soil to increase the concentration of K^+^ and Ca^2+^ in the body and improve competitiveness. Studies on *Lycopersicon esculentum* Mill. showed that inoculation with AM fungi (*Glomus clarum* and *G. intraradices*) under saline-alkali stress promoted its uptake of K^+^ and Ca^2+^ (Kong et al., 2019). Our study also showed similar results but did not show higher K^+^/Na^+^ and Ca^2+^/Na^+^ values. The reason might be that AM fungus affected the distribution of Na^+^ in *P. simonii*×*P. nigra*, and a large amount of Na^+^ was sequestered in the vacuole through certain measures. As a result, the reduction of Na^+^ in the cytoplasm makes it unnecessary for plants to accumulate more K^+^ and Ca^2+^ to balance Na^+^.

To complete the process of Na^+^ sequestration in a short period of time, plants need more transporters, and this process is regulated by gene expression. The *NHX* gene family is responsible for the compartmentalization and efflux of Na^+^ in cells. Meng and Wu (2018) analyzed the *NHX* gene family of 72 individuals of 31 species in *Populus* and found that the *Populus NHX* family contains 8 members. By the bioinformatics analysis, 8 *NHX* genes were also found in *P. simonii*×*P. nigra*. Among them, 6 genes were highly similar to *AtNHX2* and *AtNHX6*. We guess that these six genes are responsible for regulating Na^+^ compartmentalization in cells. A study showed that overexpressing the wheat *TaNHX2* gene in *Solanum melongena* L. enhanced its salt tolerance (Yarra, 2019; Yarra and Kirti, 2019). Under 200 mM salt stress, the Na^+^ concentration in the transgenic tomato was significantly higher than that of the wild type, showing a higher Na^+^ capacity. At the same time, under salt stress, the growth performance, photosynthesis, transpiration rate, and stomatal conductance of the transgenic tomato were superior to those of the wild type. Inoculation with AM fungi also produced a similar response in *P. simonii*×*P. nigra*. Compared with NMS, AM fungi increased the expression levels of Px_DH28184, Px_DH30127, Px_DH11117, Px_DH21638 and Px_DH21008 in *P. simonii*×*P. nigra* shoots under saline-alkali stress. At the same time, the results of correlation analysis also showed that these genes were related to poplar fresh weight, photosynthesis, gas exchange and ion concentration, and these parameters were also improved by AM fungal inoculation. Therefore, there is such a possibility that under saline-alkali stress, AM fungi can improve the salinity-alkali tolerance of poplar, growth performance and photosynthesis by inducing the expression of *PxNHX*s. The SOS pathway is the first saline-alkali stress signal transduction pathway established in plants (Fang et al., 2021). Px_DH21638 and Px_DH18325 are highly similar to *AtNHX7*/*SOS1*, we guess that they are involved in the SOS pathway and are responsible for regulating Na^+^ efflux in cells. *SpSOS1* can improve plant salt tolerance by regulating ion homeostasis and protecting plasma membrane (PM) from oxidative damage under salt stress (Zhou et al., 2018). In *Pistacia vera* L. cv. Ohadi, *R. irregularis* enhanced the host’s salt tolerance by inducing up-regulated expression of the *SOS1* gene (Abbaspour et al., 2021). Both Px_DH21638 and Px_DH18325 were up-regulated in aboveground and underground parts. At the same time, these two genes were also related to parameters such as fresh weight, photosynthesis, gas exchange and ion concentration of *P. simonii*×*P. nigra*. Therefore, AM fungi may also affect the salinity tolerance of *P. simonii*×*P. nigra* by regulating the expression of these genes. Interestingly, pre-mycorrhizal treatment also induced the up-regulated expression of Px_DH21638 and Px_DH18325 even without saline-alkali stress, which further illustrates the importance of pre-mycorrhizal treatment.

## 5 Conclusions

In summary, inoculation with AM fungi improved the saline-alkali tolerance of *P. simonii*×*P. nigra*. This increased tolerance may be conferred by AM fungi altering the expression of the *NHX* gene family. This study found 8 *NHX* gene family members in *P. simonii*×*P. nigra*. The expression levels of Px_DH28184, Px_DH30127, Px_DH11117, Px_DH21638, Px_DH21008, Px_DH21638 and Px_DH18325 were affected by AM fungi and changed under saline-alkali stress. On the one hand, the changes in the expression of these genes increased the concentration of Na^+^, K^+^, and Ca^2+^ in the underground part, reduced the pH and Na^+^ concentration in the rhizosphere soil, and improved the rhizosphere soil environment. On the other hand, it adjusted the distribution of Na^+^ in the aboveground part cells and increased the tissue water content of poplar. Thus reducing the toxic effect of Na+ on chloroplasts, increasing stomatal conductance, transpiration rate and net photosynthetic rate, stabilizing light and electron transport chains, and improving photosynthesis. Through these methods, the rapid growth of mycorrhizal *P. simonii*×*P. nigra* in the saline-alkali stress environment was finally realized. However, it is not enough to only study the changes within a plant at a single time, and the gene expression in plants changes dynamically. In the future, it is necessary to focus on the influence of mycorrhizae on the expression of the *NHX* gene family in *P. simonii*×*P. nigra* during the initial period of saline-alkali stress. Our findings contribute to a deep understanding of the mechanism of AMF-induced saline-alkali tolerance and lay the foundation for further improvement of plant saline-alkali tolerance.

## Data availability statement

The datasets presented in this study can be found in online repositories. The names of the repository/repositories and accession number(s) can be found in the article/**Supplementary Material**.

## Author contributions

Conceptualization, FD. Methodology and investigation, FD, YW and JT. Data curation, FD. Writing—original draft preparation, FD and TX. Writing—review and editing, TX. Supervision and project administration, MT. Funding acquisition, MT. All authors contributed to the article and approved the submitted version.
