# Supplementary material for: Arbuscular mycorrhizal fungi affect the expression of PxNHX gene family, improve photosynthesis and promote Populus simonii×P. nigra growth under saline-alkali stress
Source: Front Plant Sci. 2023 Jan 30;14:1104095. doi: 10.3389/fpls.2023.1104095 (PMC9923091; doi:10.3389/fpls.2023.1104095)
Supplement: Supplementary file 1 [file Table_1.docx]

Supplementary Table 1 *PxNHX*s quantitative primers

| Gene Name | F-primer | R-primer | Tm (℃) |
| --- | --- | --- | --- |
| Px_DH28184 | TCCTCTTCTTGCCAACGGAC | TCCGCCAAAAGTGATGGACA | 57.6 |
| Px_DH21008 | GGAGCAGCGATGGAATGACG | CTGCGCCAAACAGCATGATA | 57.6 |
| Px_DH25989 | TCTGTTGAGTGCCTGCCAAA | GCCTGGGATTTTGTTCGCAA | 56.5 |
| Px_DH11117 | AGGGGCAGGACTCGTTAGAT | TCGGAGAATTGCGCTCTGTT | 61.1 |
| Px_DH25939 | GCACCTGTGGGTGTACTAGG | CCAGCTCGAAGGGCAAGTAG | 62.7 |
| Px_DH30127 | CCTGTTCCCTGATGTGGCAT | GACAGGGACTGTGCTCGAAA | 56 |
| Px_DH21638 | TTCCTCGTCAGCTTCTTCGG | AGCAACAGTGTAAGGGACTCG | 56 |
| Px_DH18325 | TGACAAGCTGTGTGATCGCT | TTGGTTGATCACCCCCACAG | 61.1 |
| BU875027 | GGCTAATTTTGCCGATGAGA | ACGTCCATCCCTTCAACAAC | 56.5 |
| GQ253565.1 | ATTGACAGGCGGTCTGGTAAGGAA | AAACGACCAAGTGGAGGATACGCT | 56.0 |

Supplementary Table 2 Genetic characteristics of the *NHX* gene families in *P. simonii*×*P.nigra*

| Gene name | Number of amino acids (aa) | Molecular weight (kDa) | Theoretical pI | Instability index | Aliphatic index | Predicted location(s) | Transmembrane domain | Corresponding Arabidopsis Gene No. | Corresponding Arabidopsis Gene Name |
| --- | --- | --- | --- | --- | --- | --- | --- | --- | --- |
| Px_DH11117 | 544 | 60.41 | 7.3 | 38.44 | 106.25 | Vacuole | 10 | AT3G05030.1 | ATNHX2 |
| Px_DH18325 | 1145 | 126.71 | 6.21 | 33.90 | 103.54 | Cell membrane | 12 | AT2G01980.1 | ATNHX7 |
| Px_DH21008 | 536 | 59.57 | 8.76 | 42.87 | 108.56 | Vacuole | 10 | AT3G05030.1 | ATNHX2 |
| Px_DH21638 | 1147 | 127.02 | 6.49 | 40.19 | 103.78 | Cell membrane | 12 | AT2G01980.1 | ATNHX7 |
| Px_DH25939 | 318 | 35.13 | 6.7 | 33.33 | 124.50 | Vacuole | 7 | AT3G05030.1 | ATNHX2 |
| Px_DH25989 | 410 | 45.33 | 5.48 | 35.78 | 117.90 | Vacuole | 9 | AT3G05030.1 | ATNHX2 |
| Px_DH28184 | 632 | 70.77 | 8.85 | 36.74 | 111.69 | Vacuole | 11 | AT3G05030.1 | ATNHX2 |
| Px_DH30127 | 537 | 58.97 | 5.55 | 40.73 | 101.30 | Vacuole | 10 | AT1G79610.1 | ATNHX6 |

Supplementary Table 3 Two-way ANOVA of *PxNHX*s expression

| Gene | upground part | | | underground part | | |
| --- | --- | --- | --- | --- | --- | --- |
|  | *P*_AMF_ | *P*_saline-alkali_ | *P*_AMF*saline-alkali_ | *P*_AMF_ | *P*_saline-alkali_ | *P*_AMF*saline-alkali_ |
| Px_DH28184 | NS | NS | NS | ** | * | NS |
| Px_DH21008 | ** | NS | NS | ** | NS | NS |
| Px_DH25989 | ** | NS | NS | ** | NS | NS |
| Px_DH11117 | * | NS | NS | ** | NS | * |
| Px_DH25939 | * | NS | NS | NS | NS | NS |
| Px_DH30127 | NS | NS | NS | ** | * | * |
| Px_DH21638 | ** | NS | NS | ** | NS | NS |
| Px_DH18325 | NS | NS | NS | ** | NS | NS |

Note: Significant effect of two-way ANOVA analysis: “*” indicates P < 0.05, “**” indicates P < 0.01; “NS” indicates no interaction (P ≥ 0.05).
